# A case of rapid rupture of a calcified amorphous tumor observed by echocardiography

**DOI:** 10.1093/jscr/rjaf064

**Published:** 2025-02-19

**Authors:** Kentaro Shirakura, Ryohei Ushioda, Masahiro Tsutsui, Shingo Kunioka, Nobuhiro Mochizuki, Tatsuya Aonuma, Naoko Kawabata, Erika Saitoh, Naoki Nakagawa, Hiroyuki Kamiya

**Affiliations:** Department of Cardiac Surgery, Asahikawa Medical University, Midorigaoka Higashi 2-1-1-1, Asahikawa 078-8510, Japan; Department of Cardiac Surgery, Asahikawa Medical University, Midorigaoka Higashi 2-1-1-1, Asahikawa 078-8510, Japan; Department of Cardiac Surgery, Asahikawa Medical University, Midorigaoka Higashi 2-1-1-1, Asahikawa 078-8510, Japan; Department of Cardiac Surgery, Asahikawa Medical University, Midorigaoka Higashi 2-1-1-1, Asahikawa 078-8510, Japan; Department of Cardiac Surgery, Asahikawa Medical University, Midorigaoka Higashi 2-1-1-1, Asahikawa 078-8510, Japan; Department of Cardiology, Asahikawa Medical University, Midorigaoka Higashi 2-1-1-1, Asahikawa 078-8510, Japan; Department of Cardiology, Asahikawa Medical University, Midorigaoka Higashi 2-1-1-1, Asahikawa 078-8510, Japan; Department of Cardiology, Asahikawa Medical University, Midorigaoka Higashi 2-1-1-1, Asahikawa 078-8510, Japan; Department of Cardiology, Asahikawa Medical University, Midorigaoka Higashi 2-1-1-1, Asahikawa 078-8510, Japan; Department of Cardiac Surgery, Asahikawa Medical University, Midorigaoka Higashi 2-1-1-1, Asahikawa 078-8510, Japan

**Keywords:** calcified amorphous tumor

## Abstract

A calcified amorphous tumor (CAT) of the heart is a rare, non-neoplastic, intracavitary cardiac mass. Histological examination reveals the presence of calcified and amorphous fibrous material with underlying chronic inflammation. Some studies have reported that CAT typically exhibits rapid growth. However, we observed a case in which CAT unexpectedly ruptured within approximately two weeks. There was no cerebral infarction or significant valvular disease, therefore we were not sure about the indication for surgery; however considering the epidemiological possibility of CAT, we decided to operate and were able to treat the patient without complications.

## Introduction

Calcified amorphous tumor (CAT), a non-neoplastic cardiac mass composed of nodules of calcium on a background of amorphous fibrous material, was first described in 1997 [1]. Histological examination shows that it contains calcified and amorphous fibrous material with underlying chronic inflammation [1, 2]. Surgical excision is generally recommended to avoid future embolism [1–3]. Although its etiology remains unclear, several cases of CAT involving patients with end-stage renal disease (ESRD) and hemodialysis have been reported [3]. In most previously reported cases, CAT typically exhibits a characteristic of rapid growth [4]. In our case, Cardiac CATs ruptured rapidly over a relatively short period. This case report enhances our knowledge of the progress of CAT, thereby contributing to a better understanding of the clinical features of the disease.

## Case report

**Figure 1:**
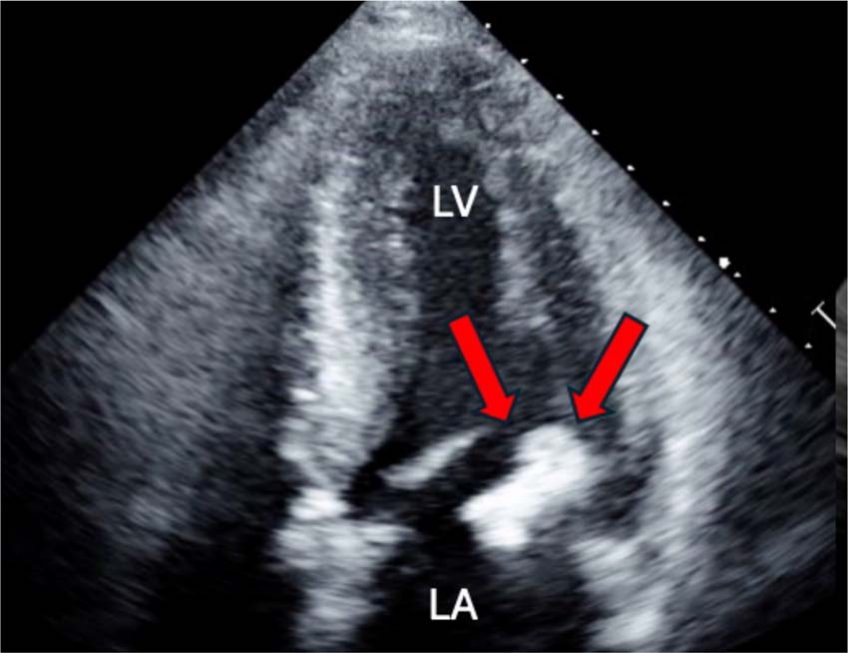
Preoperative cardio echography showing fraction EF 63%, LVDd 50 mm, LVDs. 35 mm, and a 3-cm size of mobile mass.

**Figure 2:**
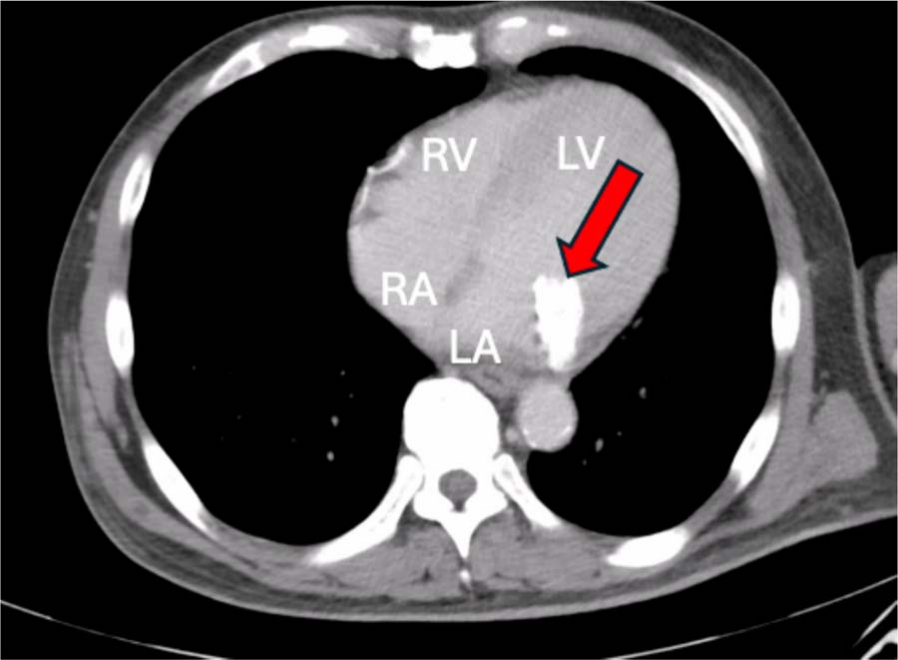
Cardiac CT showing severe MAC.

**Figure 3:**
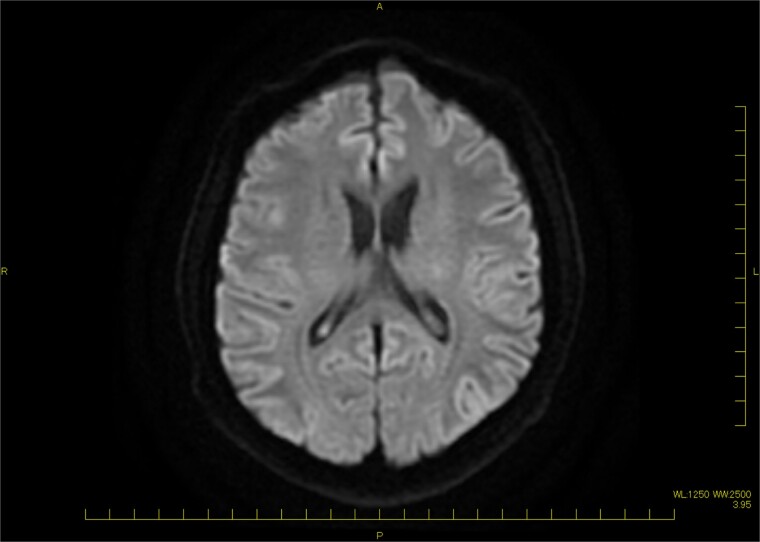
Brain MRI (DWI) showing no acute lesion.

**Figure 4:**
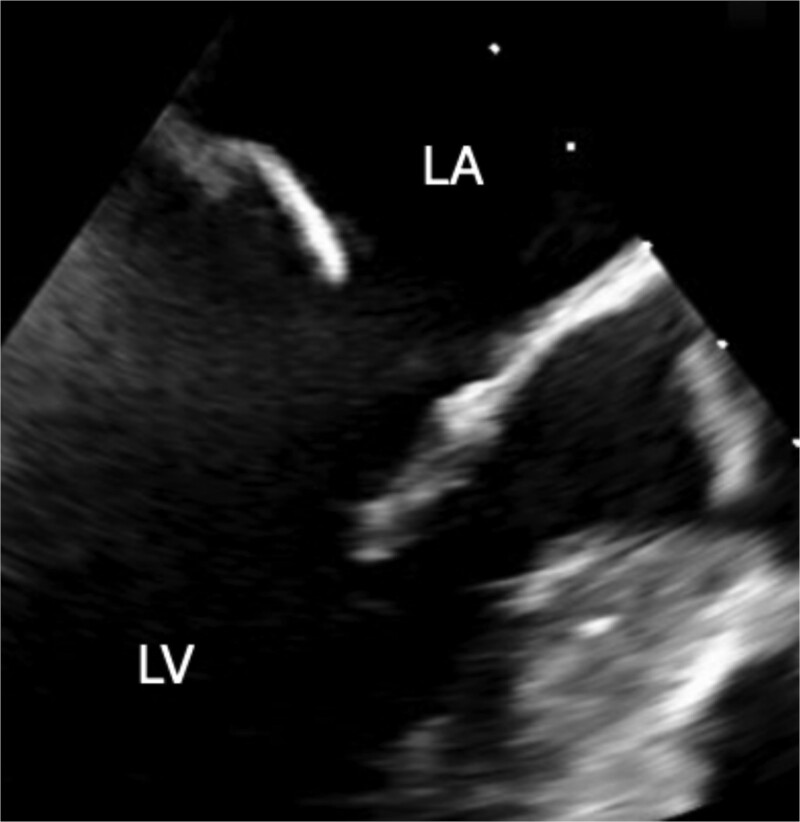
Preoperative transesophageal echocardiography showing disappearance of the mass.

**Figure 5:**
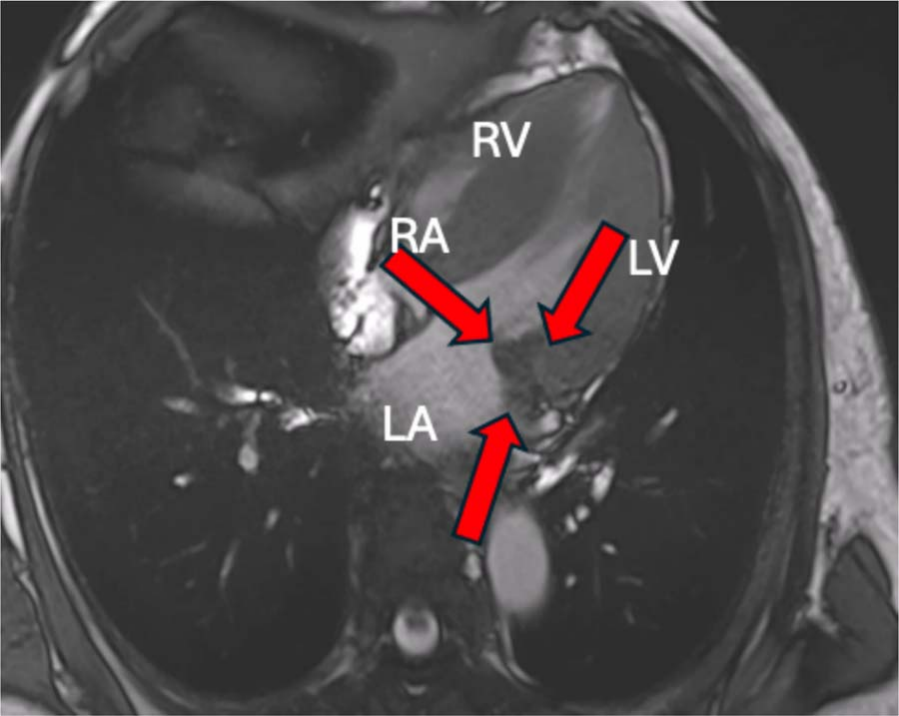
Cardiac MRI showing a hypointense mass on axial T2-weighted.

**Figure 6:**
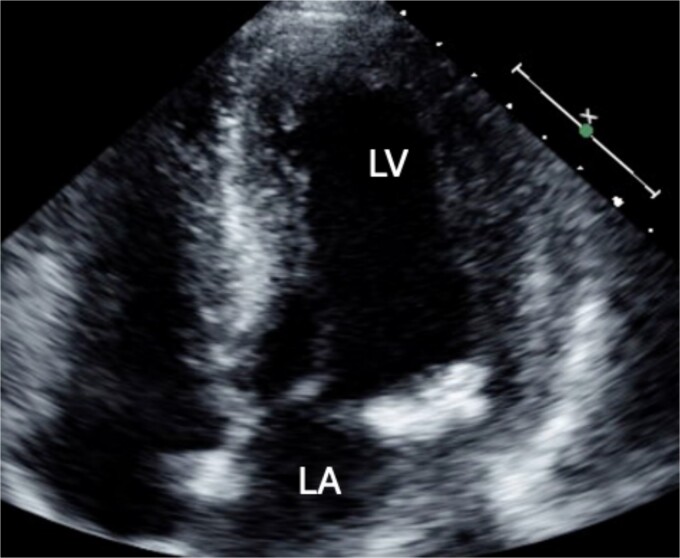
Preoperative TTE showing no mobile mass.

A 46-year-old male with a pertinent medical history of hemodialysis for chronic renal failure presented to his primary hospital in October 2023 with an undetermined fever. Following admission, he was referred to the cardiology department due to chest pain suspected of acute coronary syndrome (ACS). Transthoracic echocardiography (TTE) identified a mobile mass lesion measuring 3-cm near the posterior leaflet of the mitral valve, prompting referral to our department on suspicion of infective endocarditis (IE). Echocardiography in our hospital revealed an ejection fraction (EF) of 63%; mild mitral regurgitation (MR); left ventricular diastolic diameter (LVDd) of 50 mm; left ventricular systolic diameter (LVDs), 35 mm; and a 3 cm size of mobile mass (Fig. 1). Cardiac computed tomography (CT) showed severe mitral annular calcification (MAC) (Fig. 2). No acute lesion was seen on brain magnetic resonance imaging (MRI) (Fig. 3). On his 14th day in the hospital, operation was scheduled, however canceled due to the disappearance of the mass on transesophageal echocardiography immediately prior to surgery (Fig. 4). However, cardiac MRI in 2 weeks showed a mass lesion (Fig. 5). Therefore, we planned to perform surgery in early February 2024. Although TTE on the day before surgery did not show a mobile mass, we decided to proceed with surgery in consideration of the possibility of CAT (Fig. 6).

**Figure 7:**
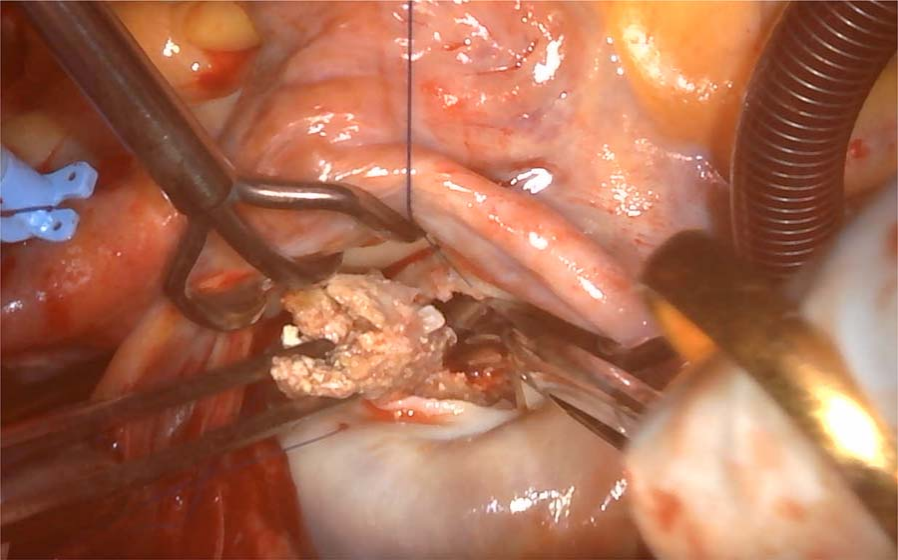
Atheromatous calcification lesion around P2 part of mitral valve.

The surgery was performed through a median sternotomy with arterial cannulation into the ascending aorta and venous cannulation into the superior and inferior vena cava. After cardioplegic arrest, a left atrial incision was made and the mitral valve was confirmed. A 2 cm calcification extending from the base of P2 to the left ventricular myocardium and a 5 mm atheromatous calcification lesion were found, which were removed as much as possible after detachment of the P2-segment from the annulus (Fig. 7). A 5 mm fistula was found on the left ventricular side of the P2 valve annulus, closed with an autologous pericardial patch. Thereafter, patch augmentation using another autologous pericardial patch was performed for the posterior leaflet. After that, an annuloplasty band (CG Future band, 32 mm; Medtronic, Minnesota) was sutured and the procedure was completed. The pathology of the intraoperative specimen revealed a diagnosis of CAT.

The patient's postoperative course was good, and he was transferred to his primary physician twenty-one days after surgery. The patient is doing very well seven months after surgery.

## Discussion

Cardiac CAT was first reported by Reynolds *et al.* in 1997 as a non-neoplastic cardiac mass that can mimic neoplasms and cause symptoms due to the obstruction or embolization of calcified fragments [1]. Notably, compared with intracavity tumor, patients with MAC-related CAT suffered from end-stage renal failure and received hemodialysis, including our case [3, 4]. It is often difficult to distinguish CAT from other cardiac tumors, vegetation, and thrombus when using single imaging modalities [4]. Regarding the treatment of CAT, surgery is typically recommended due to the high risk of sudden death and arterial embolization, even in asymptomatic patients [5–7]. In our case, we observed a brief disappearance and then a recurrence. Preoperative echocardiography did not reveal any obvious residual tissue of the mass, which had been observed two weeks before and there were no significant changes in the calcification of the MAC. And then it was rediscovered by cardiac MRI sixteen days after. The disappearance of the mass within short term interval between the initial detection of the mobile mass on TTE suggests that partial rupture of the mass may have occurred. In a case that the tumor disappears on echocardiography, cardiac MRI may help diagnosis. It is reported that CATs show low signal intensity on T1- and T2-weighted images with no contrast enhancement [8]. There have been no prospective epidemiological studies on CAT, and the etiology of a cardiac CAT remains still unclarified. Further research is needed on diagnostic methods and appropriate treatment.

## Conclusion

Cardiac CATs can rupture rapidly over a relatively short period, as demonstrated in this case. When the mobility is lost, it is difficult to distinguish from MAC on echocardiography, and cardiac MRI may be useful. In our case, there was no cerebral infarction or significant valvular disease, therefore we were not sure about the indication for surgery; however considering the epidemiological possibility of CAT, we decided to operate and were able to treat the patient without complications. Although clear indications for surgery have not been established, we believe that surgery should be performed in epidemiologically doubtful cases.
